# Genetic Diversity and Core Germplasm Identification in *Penaeus japonicus* Using Whole-Genome Resequencing

**DOI:** 10.3390/ani15182759

**Published:** 2025-09-22

**Authors:** Dingyuan Zhang, Jikang Shentu, Weijian Liu, Yanxia Wang, Minjun Zhu, Zhiming Yang, Liegang Si

**Affiliations:** Ningbo Ocean and Fisheries Research Institute, Ningbo 315211, China; 18358052655@163.com (D.Z.);

**Keywords:** *Penaeus japonicus*, population genetics, core collection, resequencing

## Abstract

The kuruma shrimp (*Penaeus japonicus*), a globally high-value aquaculture species, faces critical challenges in sustainable development due to genetic diversity degradation and declining disease resistance. This study employed whole-genome resequencing (WGRS) to systematically assess genetic diversity, population structure, and core germplasm characteristics across 20 geographically distinct populations from Zhejiang, Fujian (China), and introduced Japanese stocks. Using 343.40 Gb of high-quality sequencing data (average depth: 12.44×), we identified 9,146,248 single nucleotide polymorphisms (SNPs), with 6.32% located in exon regions, while intergenic (56.75%) and intronic regions (30.99%) showed the highest polymorphism density. Principal component analysis (PCA) and phylogenetic tree construction revealed two major clades: Fujian (FJ) and Japan-introduced (RB) populations clustered closely due to shared artificial breeding backgrounds, whereas Zhejiang (XS) and Fujian (LS) populations displayed genetic heterogeneity driven by adaptive divergence. Core germplasm screening via the CoreHunter algorithm selected four representative individuals (FJ4-M, LS1-M, XS1-M, XS6-M), with the modified Rogers’ distance (0.34) and allele coverage (0.93) confirming effective preservation of original genetic diversity. This study provides genomic insights and technical frameworks for germplasm conservation, precision breeding, and genetic improvement in *P. japonicus*.

## Simple Summary

Kuruma shrimp (*Penaeus japonicus*) aquaculture faces sustainability threats from reduced genetic diversity and disease resistance. Traditional genetic markers offer limited insight, and breeding worsens genetic homogenization. This study used whole-genome resequencing (WGRS) to analyze genetic diversity, structure, and core germplasm across 20 populations from Zhejiang, Fujian (China), and introduced Japanese stocks. By generating 343.40 Gb of high-quality data (avg. depth 12.44×), researchers identified 9,146,248 SNPs, with most located in intergenic (56.75%) and intronic (30.99%) regions. Population analysis revealed that Fujian (FJ) and Japan-introduced (RB) populations clustered closely due to shared artificial breeding backgrounds, whereas Zhejiang (XS) and Fujian (LS) populations displayed genetic heterogeneity driven by adaptive divergence. Core germplasm screening selected four representative individuals (FJ4-M, LS1-M, XS1-M, XS6-M), effectively preserving original diversity (allele coverage 0.93). This provides genomic resources and a framework for germplasm conservation and breeding improvement in *P. japonicus*.

## 1. Introduction

The kuruma shrimp (*Penaeus japonicus*), also known as the Japanese tiger prawn, is recognized as a strategically significant aquaculture species globally and is one of the economically important species driving the development of the global shrimp industry due to its rapid growth rate, strong environmental adaptability, and potential for high-density cultivation [1]. Despite the dominant global production of *Litopenaeus vannamei*, reaching 6.8 million tons in 2023, *P. japonicus* retains a critical position in premium markets across East Asia and the Mediterranean region owing to its unique market niche and nutritional value. Characterized by high protein and a low-fat content, its nutritional profile not only fulfills human dietary requirements but also plays a pivotal role in regional food security and the advancement of the blue economy [2]. Since the 1970s, aquaculture technologies for *P. japonicus* have been progressively refined, leading to the establishment of large-scale industrial systems in coastal regions of Japan, China, and Australia. However, with the intensification of farming practices, challenges such as germplasm degradation, frequent disease outbreaks, and reduced environmental resilience have become increasingly prominent, posing significant threats to sustainable industry development [3,4]. It has been demonstrated that prolonged artificial selection and inbreeding have led to marked reductions in genetic diversity within cultured populations, exacerbating genetic bottleneck effects and compromising stress resistance and growth performance [5,6]. Therefore, a systematic evaluation of genetic diversity and population structure in *P. japonicus* is deemed imperative for optimizing germplasm management, guiding genetic improvement, and ensuring the sustainability of aquaculture practices.

Genetic diversity, regarded as the fundamental basis for species adaptation to environmental fluctuations and the maintenance of evolutionary potential, serves as the theoretical foundation for the design of genetic breeding strategies [7]. Elucidation of population genetic structure and diversity distribution patterns is essential for formulating efficient breeding programs, mitigating inbreeding depression, and rationally utilizing wild germplasm resources [8]. Traditionally, genetic studies on *P. japonicus* have predominantly relied on microsatellite markers or mitochondrial gene fragments [5,9,10]; however, the limited genomic coverage of these methods has constrained comprehensive insights into the genetic regulation of complex traits. With the widespread adoption of high-throughput sequencing technologies [11,12], whole-genome resequencing (WGRS) has emerged as a powerful tool for mining single-nucleotide polymorphisms (SNPs), assessing population genetic differentiation, and detecting selection signatures [13]. For instance, Wang et al. [14] identified 37 million SNPs via WGRS, revealing pronounced stratification among six *Litopenaeus vannamei* breeds. Similarly, Bao et al. [15] uncovered candidate genes of *L. vannamei* associated with rapid growth traits through genome-wide analysis, while Sui et al. [16] utilized WGRS to quantify genetic parameters for growth traits and resistance to white spot syndrome virus (WSSV) in *L. vannamei*. Nevertheless, there are still few comparative studies on the genetic architecture of distinct geographic populations and commercially selected strains of *P. japonicus*, particularly regarding systematic elucidation of genetic divergence patterns among Asian aquaculture populations. Since its introduction to Asia in the 1980s, the genetic structure of *P. japonicus* is hypothesized to have undergone significant alterations due to introduction history, breeding strategies, and environmental adaptation [17]. Existing evidence suggests moderate genetic differentiation among geographically distinct *P. japonicus* populations [5]. However, gene flow resulting from large-scale farming may obscure inherent genetic patterns. Consequently, the integration of multi-population and cross-regional genomic data is imperative for precisely delineating genetic structure, tracing germplasm dispersal pathways, identifying unique genetic resources, and formulating differentiated conservation strategies.

This study employed whole-genome resequencing technology to obtain high-density SNP markers for assessing genetic diversity levels, genetic differentiation, and population structure across distinct populations of *P. japonicus*. The research further elucidated potential impacts on artificial breeding programs. These conclusions provide a theoretical foundation for germplasm conservation, precision breeding, and the sustainable development of *P. japonicus* aquaculture.

## 2. Materials and Methods

### 2.1. Sample Collection and DNA Extraction

In this study, the *P. japonicus* populations were from 11 populations in Qifeng, Zhejiang, 4 populations in Fujian, 3 populations in Shipu, Zhejiang, and 2 introduced populations from Japan. Specific information is shown in Table 1. The populations from Zhejiang and Fujian were sourced from local wild fishing, while those from Japan were introduced from commercial breeding lines. These populations were all raised at the Hengma Base of Ningbo Marine Fisheries Research Institute, and the egg production of each population was recorded. In total, 50 offspring were randomly captured, and their weight was recorded for each month from January to June to ensure consistency in the impact of the environment on different populations and maintain consistent breeding conditions for each population. In addition, ten individuals were randomly selected from each population, and muscle tissue was isolated from fresh samples, stored in 75% ethanol, and extracted using a marine animal tissue genomic DNA extraction kit (DP324, Tiangen Biochemical Technology (Beijing) Co., Ltd., Beijing, China). The quality of the extracted DNA was detected using 1.2% agarose gel electrophoresis, and the concentration of DNA was determined using a spectrophotometer (QUAWELL, San Diego, CA, USA). High-quality DNA was diluted to 50 ng/mL and stored at −20 °C.

### 2.2. Growth Performance Analysis

The reproductive capacity of shrimp is a core biological indicator reflecting the reproductive potential of female shrimp, and it is measured by the ratio of egg quantity per unit weight. The weight gain rate (WGR) and the specific growth rate (SGR) were selected as the growth performance index, which were calculated according to the following formulas: WGR (%) = 100 × (final total weight − initial total weight)/initial total weight; SGR (%/d) = 100 × ((ln(final body weight) − ln(initial body weight))/feeding days.

### 2.3. Sequencing and Sequence Alignment

Ten DNA samples from each population were mixed in equal mass, and then the mixed DNA samples were sent to Novogene Biotechnology Co., Ltd., Beijing, China, for sequencing. High-quality DNA samples were randomly broken into 350 bp fragments using ultrasonic fragmentation technology. The resequencing library was constructed using the TruSeq Library Construction Kit (Illumina, Inc., San Diego, CA, USA) and strictly followed the recommended reagents and consumables in the instruction manual. The constructed library was sequenced using the Ilumina HIiSeq 2500 platform. The raw data were subjected to quality control screening, filtering out adapter sequences, low-quality sequences (MQ ≥ 6), and short fragments (length < 500 bp) to obtain clean reads after quality control. BWA v 0.7.17 software was used to compare clean reads with the *P. japonicus* genome (GenBank: GCA 017312705.1) [18], and SAMtools v1.17 software was used to calculate the alignment rate and coverage [19].

### 2.4. Mutation Detection and Structural Annotation

The GATK v3.8 software was used to detect SNP mutations, with the following filtering parameters: SNP: QD < 2.0, MQ < 40.0, FS > 60.0, MQRankSum < −12.5, ReadPosRankSum < −8.0 [20]. We performed quality control on the GATK-derived SNP set. This step aimed to remove potential bias and noise in the analysis. The bias and noise were caused by SNPs with high missing rates or rare variants with low MAF. We excluded SNPs with a site missing rate greater than 5%. We also removed rare variants with a minor allele frequency (MAF) less than 1%. All subsequent core analyses were based on this filtered dataset. The final dataset had a missing rate of ≤5% and a MAF of ≥1%. Additional criteria followed GATK Best Practices, ensuring high-confidence variant calls for downstream analyses. Lumpy v0.3.1 software was used to detect structural variations, and CNVnator v0.3.3 software was used to detect copy number variations [21]. ANNOVAR (2018Apr16) and SnpEff v5.3 were used to annotate the positions and functions of SNPs, SVs, and CNVs [22,23].

### 2.5. Analysis of Population Genetics

After SNP detection, the individual SNPs obtained could be used to calculate genetic distances between populations. Treebest-1.9.2 software was used to calculate the distance matrix, and based on this, a phylogenetic tree was constructed between 20 *P. japonicus* populations using the neighbor-joining method, and bootstrap values were obtained through 1000 calculations [24]. Nodes with bootstrap support values of ≥70% were considered to have reliable statistical support, indicating that the population divergence represented by that branch was significant. In the final tree, individuals from the same geographic origin or with shared breeding history were expected to cluster into monophyletic groups with high bootstrap values. Principal component analysis (PCA) is a purely mathematical operation method that can select a smaller number of important variables by linearly transforming multiple related variables [25]. PCA was conducted using GCTA v1.93 software, based on the degree of SNP differences in individual genomes; individuals were clustered into different subgroups according to principal component analysis based on different trait characteristics [26]. In the plot, individuals with similar genetic backgrounds tended to aggregate closely in space, forming distinct clusters. The extent of genetic differentiation between populations was visually assessed based on the spatial distance and separation between different clusters. Individuals belonging to the same cluster were regarded as having low genetic differentiation, while clear separation into distinct clusters indicated significant genetic divergence between populations. The results of PCA and phylogenetic analysis corroborated each other, collectively defining the population genetic structure. VCFtools v 0.1.16 software was used to calculate the nucleotide diversity of various groups [27]. Admixture software was used to construct population genetic structure and population lineage information [28].

### 2.6. Core Germplasm Analysis

The germplasm of a species refers to the genetic material passed down from parents to offspring during the genetic process. The software CoreHunter 3.2.1 algorithm “*MR*” was used to perform core germplasm analysis, setting the proportion of the core sample group to 20% of all samples [29]. *MR* is a genetic distance calculation method based on the Wrighter–Fisher model. For a marker number “*m*”, “*p_ij_*” represents the frequency of the “*j*”-th allele at the “*i*”-th locus in population “*p*”, and “*q_ij_*” represents the frequency of the “*j*”-th allele at the “*i*”-th locus in population “*q*”. “*a_i_*” is the number of alleles at the “*i*”-th locus. The specific calculation formula is as follows:

$$MR\;=\;\sqrt{\frac{1}{2m}\sum_{i=1}^{m}\sum_{j=1}^{a_{i}}{\left(p_{ij}-q_{ij}\right)}^{2}}$$

## 3. Results

### 3.1. Analysis of Growth Performance Results

**Figure 1:**
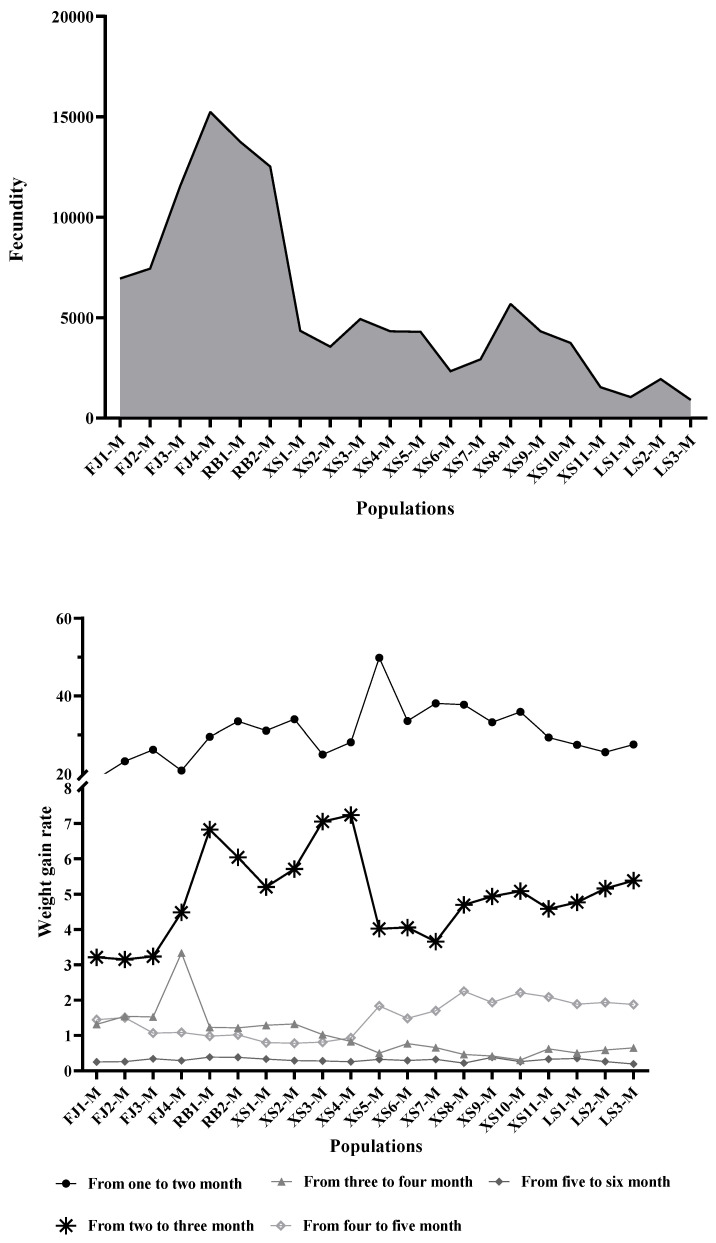

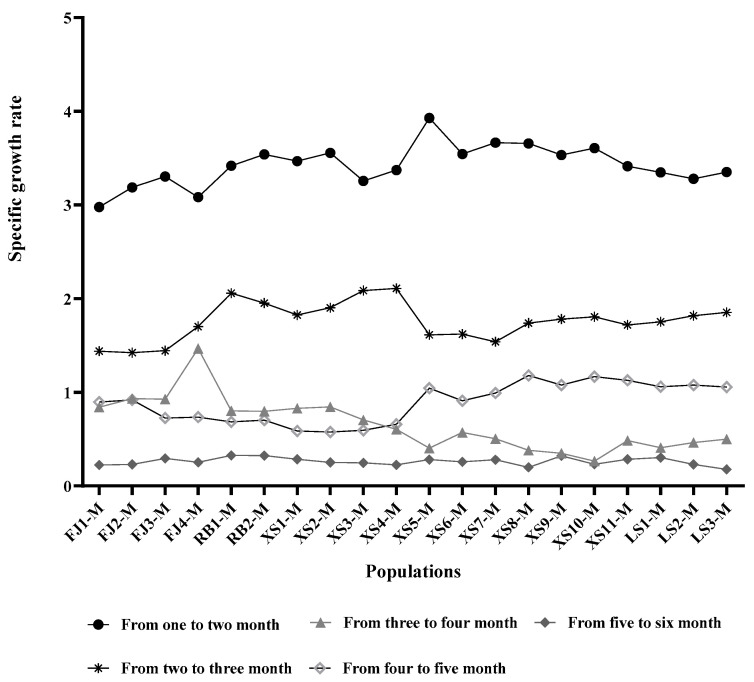
Growth performance parameters of 20 *P. japonicus* populations.

This study conducted a comparative analysis of the growth performance of the 20 *P. japonicus* populations during the growth period from January to June. The results showed that there were significant differences in fecundity among the 20 *P. japonicus* populations, ranging from 913.92 to 15245.75. Among them, there was no statistically significant difference between the FJ and RB populations (*p* > 0.05), but there was an extremely significant difference between the FJ and XS and LS populations (*p* < 0.01). There were also extremely significant differences between the RB population and both the XS and LS populations (*p* < 0.01). There was no significant difference between the XS and LS populations (*p* > 0.05). Furthermore, an analysis was conducted on the weight gain rate and specific growth rate among the four FJ populations, two RB populations, XS1-M, XS2-M, and XS3-M populations. It was found that both the weight gain rate (WGR) and the specific growth rate (SGR) showed a gradual decline with increasing age. In contrast, the WGR and SGR indicators of the remaining 13 experimental populations were significantly higher in the 4–5-month age stage than those in the 3–4-month age stage (*p* < 0.05), showing typical stage-specific growth acceleration characteristics (Figure 1).

### 3.2. Quality Control and Evaluation of Data, and Comparison with Reference Genome

Based on the reference genome of *P. japonicus*, 20 samples were subjected to whole-genome resequencing analysis. According to quality control statistics, the total data volume of Raw Base was 360.74 Gb. After strict filtering and processing, a high-quality Clean Base data volume of 343.40 Gb was obtained, with high sequencing quality (Q20 ≥ 96.22%, Q30 ≥ 91.40%). The average effective rate was 95.20%, and the average GC content was 41.47%. Its distribution characteristics were in line with the expected eukaryotic genome (Table S1). Furthermore, comparative analysis with the reference genome showed a high degree of consistency between the sequencing data and the reference genome. After correcting for genomic gap regions, the average sequencing depth reached 12.44 × (Table S2). The efficiency of inter-sample comparison remained stable in the range of 92.41–93.97%. Genomic coverage analysis showed that effective genomic regions with ≥1 × coverage accounted for 69.52–82.73%, while core genomic regions with ≥4 × coverage accounted for 47.16–57.74%. The above system evaluation indicators confirmed the reliable quality of the sequencing library constructed in this experiment; the obtained data had high biological credibility and could provide a solid foundation for subsequent population genetics analysis.

### 3.3. Genetic Structure

**Figure 2:**
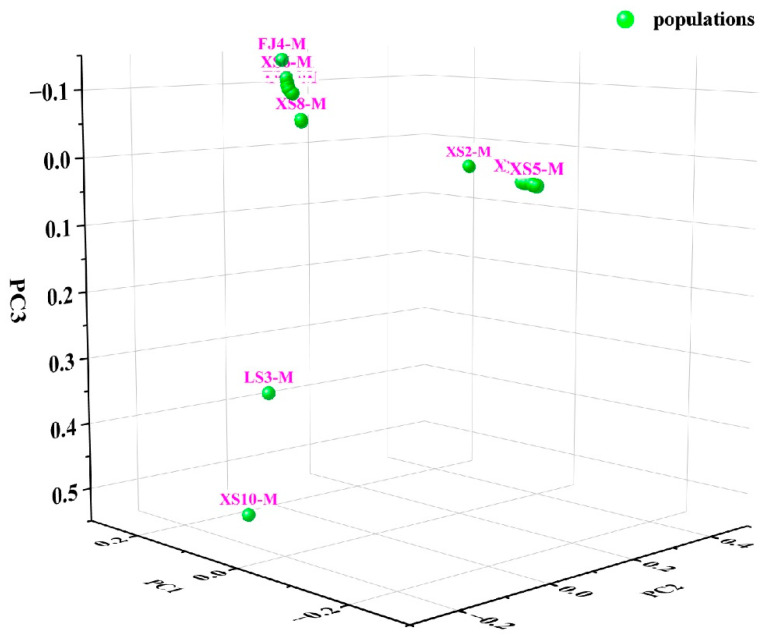
PCA three-dimensional cluster diagram of the 20 *P. japonicus* populations.

**Figure 3:**
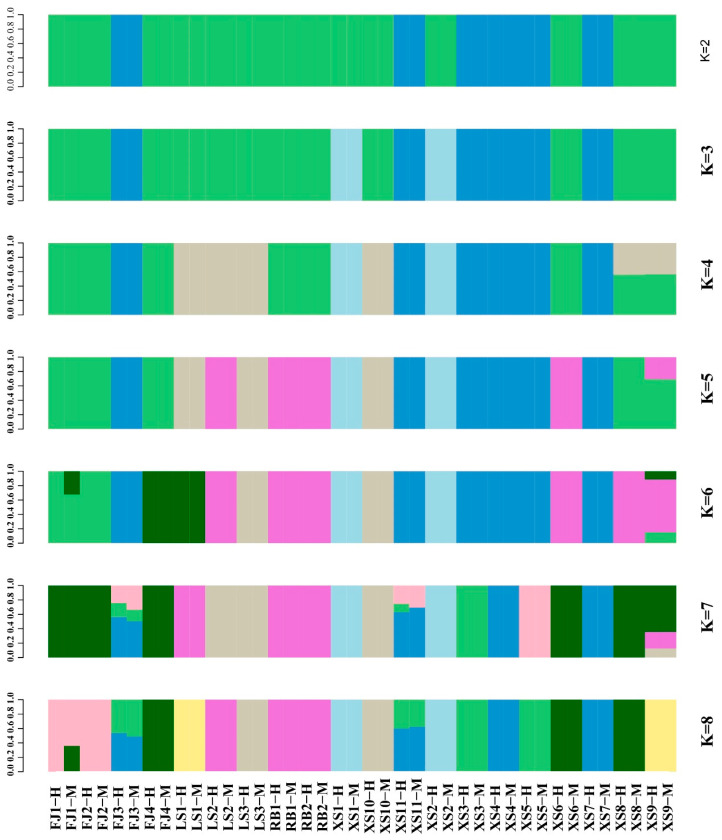
Population Structure of 20 *P. japonicus* populations.

The population genetic structure analysis strategy of the maximum likelihood method was adopted in this study, and Admixture v1.3 software was used to classify 20 samples of *P. japonicus* into taxonomic groups. PCA analysis was conducted on the resequencing data of 20 *P. japonicus* populations, and it was found that they were mainly divided into two clusters (Figure 2). A genetic clustering model constructed from whole-genome resequencing data showed that when the estimated range of the genetic clustering parameter K was set to 2–8, the cross-validation error decreased to its minimum at K = 2, indicating that the model had the optimal clustering resolution under this parameter (Figure 3). Furthermore, a linkage disequilibrium analysis was conducted, which provided a theoretical basis for analyzing genomic selection signals under natural selection and artificial domestication pressures, and particularly provided favorable guidance for creating diversified germplasm resources.

### 3.4. Localization Analysis, Detection, and Annotation of SNPs

Based on sequencing data from 20 genomic DNA libraries of *P. japonicus*, this study detected and annotated these SNPs and identified a total of 9,146,248 SNPs. The genomic distribution characteristics were as follows: In terms of gene structure region distribution, a total of 578,212 SNPs were annotated in the exon region, including 122,233 variant sites located in the 3′-UTR region and 64,768 variant sites located in the 5′-UTR region. It is worth noting that 999 mutation sites that caused genes to acquire stop codons and 125 mutation sites that caused genes to lose stop codons were detected. In total, 306,354 synonymous mutations and 120,879 non-synonymous mutations were identified in the coding sequence, and 12,834 potential regulatory variations with unknown functions were discovered. Non-coding region variations showed significant enrichment features: 2,836,035 SNPs were detected in intronic regions (accounting for 30.99% of the total variation), including 754 potential splice variants located in conserved splice site regions (intron exon boundary ± 2 bp). The intergenic region showed the highest polymorphism density, with a total of 5,192,740 SNPs annotated (56.75%). In the cis-regulatory element region, 1536 SNPs were located in the upstream 1 kb promoter region of genes, of which 5744 loci were simultaneously mapped to the downstream 1 kb regulatory region of adjacent genes. Analysis of base substitution types showed a ratio of 1.74:1 (5,806,141 vs. 3,340,107) between transitions and subversions, which is consistent with eukaryotic genome mutation preference characteristics. It is worth noting that the exon region only accounted for 6.32% of the total SNP library (approximately 1% of which was functionally annotated), which is consistent with the evolutionary feature of high repeat sequence proportion in crustacean genomes (Table 2).

### 3.5. Construction of the Phylogenetic Tree

**Figure 4:**
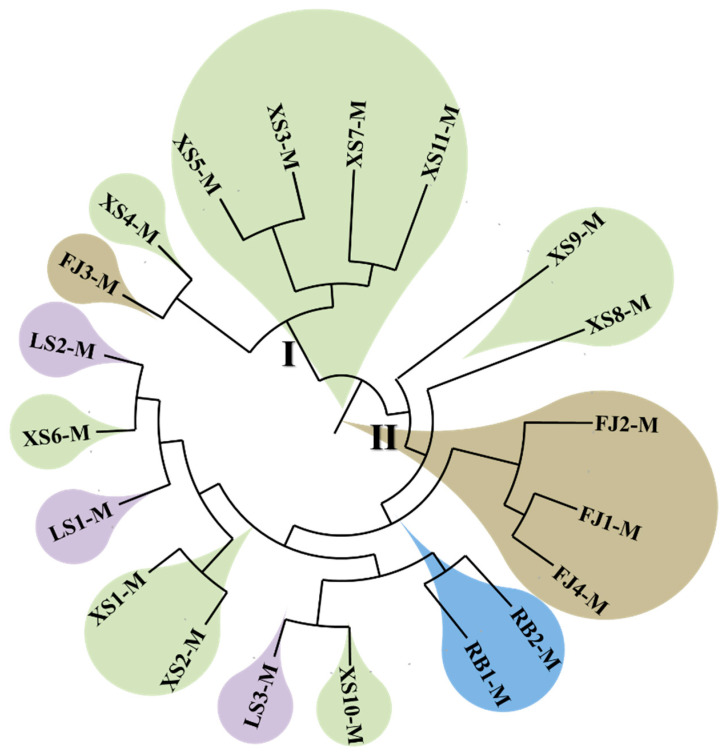
Phylogenetic tree of 20 *P. japonicus* populations based on SNP construction.

After SNP detection, the individual SNPs obtained were used to calculate the distance between populations. Based on this, a phylogenetic tree was constructed using the neighbor-joining method (Figure 4), which was mainly divided into two branches. The first branch was mainly composed of the XS population, while only one sample from the FJ population was clustered into branch I. In the other major branch, all four populations were distributed, with the FJ and RB populations clustered more densely, while the LS and XS populations clustered more evenly. Although the RB population was concentrated into one branch, its clustering with the LS and XS populations also showed the weak regional characteristics of the RB population.

### 3.6. The Construction and Evaluation of Core Germplasm

The construction of a core germplasm bank provided optimization strategies for the systematic preservation, scientific evaluation, and efficient utilization of germplasm resources. The “MR” algorithm in CoreHunter 3.2.1 software was used for core germplasm analysis in this study. The core set size was set to 20% of the original sample size (*n* = 4), and four representative samples, FJ4-M, LS1-M, XS1-M, and XS6-M, were ultimately selected. Genetic diversity analysis showed that the genetic distance indicators modified the Rogers’ distance (0.34), and the Cavalli–Sforza–Edwards distance (0.37) of the core germplasm bank remained at a high level while having a low proportion of invalid alleles (0.07) and a high allele coverage (0.93) (Table 3). It is worth noting that, based on the results of systematic clustering analysis, the RB population was not included in the core set, further confirming that this population lacks significant regional specificity. Comparative analysis of genetic parameters (Table 4) showed that the main genetic diversity parameters of the core germplasm bank showed a slight downward trend compared to the original population, while the heterozygosity balance ratio slightly increased, but the differences did not reach statistical significance levels (*p* > 0.05). Comprehensive analysis results showed that the constructed core germplasm library not only effectively maintained the genetic characteristics of the original germplasm resources but also achieved a balanced distribution of genetic diversity, especially for the reasonable characterization of non-regional specific populations.

## 4. Discussion

*P. japonicus* held an important position in global aquaculture [1], with its rapid growth rate, strong environmental adaptability, and high-density aquaculture potential making it a key species driving the development of the global shrimp industry. However, the differences in breeding varieties and technologies, genetic degradation, and disease outbreaks became increasingly prominent, posing a significant threat to the sustainable development of the industry. For example, studies by [30,31] showed that long-term artificial selection and inbreeding significantly reduced genetic diversity within aquaculture populations, exacerbated genetic bottleneck effects, and weakened resistance to stress and growth performance. In addition, white spot syndrome virus (WSSV) infection was one of the main pathogens affecting the health of *P. japonicus* [32,33]. Therefore, it was particularly important to conduct a detailed genetic background investigation of *P. japonicus* in the early stages of genetic breeding. Here, this study first compared and analyzed the growth performance of 20 *P. japonicus* populations during the growth period from January to June. Based on the analysis results, we speculated that the similarity between the FJ and RB populations may be due to their shared genetic background or introduction history, resulting in similar reproductive performance. The XS and LS populations may have adapted to specific ecological environments through long-term natural selection, thereby exhibiting higher reproductive capacity. This difference highlighted a trade-off under selective pressures: commercial breeding aimed at enhancing growth and disease resistance appeared to reduce the reproductive output in some populations, likely due to resource allocation favoring growth over reproduction. Notably, 11 *P. japonicus* populations (i.e., the XS4 to XS11 and LS1 to LS3 populations) showed abnormally high growth rates in the fourth to fifth months, which may be related to targeted selection of rapid growth traits during the breeding process, but their long-term adaptability still needs to be verified.

Genetic diversity is considered the fundamental basis for species to adapt to environmental fluctuations and maintain evolutionary potential, as well as the theoretical cornerstone for designing genetic breeding strategies [7]. Therefore, the systematic evaluation of the genetic diversity and population structure of *P. japonicus* is crucial for optimizing germplasm management, guiding genetic improvement, and ensuring the sustainability of aquaculture practices. This study systematically analyzed the genetic structure and SNP distribution characteristics of 20 populations using whole-genome resequencing (WGRS) technology. A total of 9,146,248 SNP loci were identified, of which approximately 1% were located in exon regions, while the rest were mainly distributed in intergenic and intronic regions. Analysis of nucleotide diversity (π), PIC, and heterozygosity (Ho, He) showed that the genetic diversity of the original germplasm population was at a high level, indicating that these populations have the potential for genetic breeding. Through principal component analysis (PCA), a phylogenetic tree (NJ tree), and mixed model (Admixture) analysis, this study found that the population of *P. japonicus* could be divided into two major branches. Among them, the first major branch was mainly dominated by the XS population, while the other branch had four populations distributed. The FJ and RB populations were clustered together, while the LS and XS populations were evenly distributed. This divergence may have originated from differences in introduction history and artificial breeding strategies. Studies on aquatic animals such as the black tiger shrimp (*Penaeus monodon*), mantis shrimp (*Oratosquilla oratoria*), and loach (*Misgurnus anguillicaudatus*) also showed that their population structures were influenced by their biological learning and local gene flow, exhibiting regional clustering (branches in Hunan and Hubei provinces, China) [34,35,36]. It is worth noting that the RB population was not included in the final core germplasm set. This result likely reflected that the population had a relatively single genetic background and lacked significant geographic population specificity due to frequent introductions and artificial breeding in history. Genome-wide SNP analysis provided further support for this inference: the genetic distance between the RB population and other populations (especially the FJ population) was relatively low, and the frequency of private alleles was significantly lower than that of wild or local populations such as XS and LS, which further suggested that their genetic background may have experienced genetic bottlenecks caused by artificial selection. This phenomenon has also been reported in other farmed crustacean species. For example, in the giant tiger prawn (*Penaeus monodon*), cultured populations subjected to multiple generations of selection also exhibited reduced genetic diversity and a loss of population-specific characteristics [35]. Similarly, in Pacific white shrimp (*Litopenaeus vannamei*), intensive commercial breeding led to genetic homogenization in some introduced populations, making it difficult for them to form independent branches in phylogenetic analyses [14]. The results of this study further emphasize that in the construction of core collections, special attention should be paid to populations that may have lost genetic distinctiveness due to historical introductions and breeding, to prevent them from masking or diluting the genetic characteristics of native local germplasms.

In order to continuously protect and efficiently utilize germplasm resources, ref. [37] proposed the concept of core collection (CC), which selects germplasm resources in a hierarchical manner to maximize the preservation of the genetic diversity of the original population. This study screened four core germplasms (FJ4-M, LS1-M, XS1-M, XS6-M) using the CoreHunter algorithm, and their genetic parameters (such as the HWB ratio and allele coverage) showed that they could effectively preserve the genetic characteristics of the original population. However, the genetic diversity of the core germplasm was slightly lower than that of the original population, which is consistent with the phenomenon of declining genetic diversity in most cultured animal and plant populations [38,39,40]. This result may have reflected a preference for individuals with extreme phenotypes in the core germplasm screening process or insufficient coverage of rare alleles in germplasm preservation strategies. Previous studies on the leopard coral grouper (*Plectropomus leopardus*) further revealed the complexity of core germplasm management: due to mixed parental sources and a lack of systematic breeding, the genetic differentiation of its population was mainly driven by ancestral mutations and adaptive evolution [41]. In comparison, the construction of the core germplasm of *P. japonicus* relied more on the combination of geographical sources and phenotype data. But, in the future, genome selection (GS) technology must be introduced, combined with genome-wide association analysis (GWAS) to screen alleles related to important economic traits such as disease resistance and growth rate, in order to improve the comprehensive performance of core germplasm [42,43,44]. Specifically, the Japanese shrimp farming population (RB) in this study did not exhibit significant regional differentiation, and its core germplasm construction (FJ4-M, LS1-M, etc.) did not include the RB population, indicating that the RB population may lack genetic uniqueness due to frequent gene exchange. This result further suggests the need to balance gene flow and genetic diversity protection in germplasm resource management in order to avoid homogenization of local germplasm resources caused by excessive introduction. Overall, the construction of core germplasm provided direct guidance for the preservation and utilization of *P. japonicus* germplasm and future genetic breeding.

## 5. Conclusions

Based on whole-genome resequencing (12.44× depth) of 20 *P. japonicus* populations, we identified 9.14 million SNPs, predominantly in intergenic (56.75%) and intronic regions (30.99%). Population structure analysis revealed two distinct clades: the Fujian (FJ) and Japan-introduced (RB) populations clustered closely due to shared breeding history, while the Zhejiang (XS) and Fujian (LS) populations exhibited adaptive divergence. A core germplasm of four individuals (FJ4-M, LS1-M, XS1-M, XS6-M) preserved 93% of the original genetic diversity (allele coverage = 0.93; modified Rogers’ distance = 0.34). Exclusion of the RB populations from the core set indicates reduced genetic uniqueness from frequent gene flow. This study provides a genomic framework for germplasm conservation and precision breeding.

## Figures and Tables

**Table 1:** Summary of sample name and source information.

Serial Number	Sample Name	Sample Source
1	FJ1-M	Fujian, China
2	FJ2-M	Fujian, China
3	FJ3-M	Fujian, China
4	FJ4-M	Fujian, China
5	RB1-M	Japan
6	RB2-M	Japan
7	XS1-M	Qifeng, Zhejiang, China
8	XS2-M	Qifeng, Zhejiang, China
9	XS3-M	Qifeng, Zhejiang, China
10	XS4-M	Qifeng, Zhejiang, China
11	XS5-M	Qifeng, Zhejiang, China
12	XS6-M	Qifeng, Zhejiang, China
13	XS7-M	Qifeng, Zhejiang, China
14	XS8-M	Qifeng, Zhejiang, China
15	XS9-M	Qifeng, Zhejiang, China
16	XS10-M	Qifeng, Zhejiang, China
17	XS11-M	Qifeng, Zhejiang, China
18	LS1-M	Shipu, Zhejiang, China
19	LS2-M	Shipu, Zhejiang, China
20	LS3-M	Shipu, Zhejiang, China

**Table 2:** SNP detection statistics and annotation results.

Category		Number of SNPs
Upstream		153,651
UTR3		122,233
UTR5		64,768
UTR5; UTR3		79
Exonic	Stop gain	999
	Stop loss	125
	Synonymous	306,354
	Non-synonymous	120,879
	Unknown	12,834
Intronic		2,836,035
Splicing		754
Downstream		120,999
Upstream/downstream		5744
Intergenic		5,192,740
Other		208,054
ts		5,806,141
tv		3,340,107
ts/tv		1.738
Total		9,146,248

Note: Total: the total number of SNPs; Upstream: 1 Kb upstream region of the gene; Exonic: the mutation is located in the exon region; Stop gain: a mutation that causes a gene to acquire a stop codon; Stop loss: a mutation that causes a gene to lose its stop codon; Synonymous: synonymous variation; Nonsynonymous: non synonymous variation; Intronic: the mutation is located in the intron region; Splicing: the mutation is located at the splicing site (2 bp near the exon/intron boundary in the intron); Downstream: 1 Kb region downstream of the gene; Upstream/downstream: the upstream 1 Kb region of a gene, which is also located downstream 1 Kb of another gene; Intergenic: the variation is located in the intergenic region; ts: transitions, transformation; tv: transversions, Subversion; Ts/tv: the ratio of conversion to transposition; Total: the total number of SNP sites.

**Table 3:** Evaluation of genetic diversity of core germplasm in core populations.

Method	Core Collection	MR	MRmin	CE	CEmin	PN	CV
CoreHunter (20%)	4	0.34	0.21	0.37	0.23	0.07	0.93

Note: MR: modified Rogers’ distance; CE: Cavalli–Sforza and Edwards distance; PN: proportion of non-informative alleles; CV: coverage of alleles.

**Table 4:** Population genetics analysis of original and core populations.

Group Name	*Na*	*Ne*	*PIC*	*Pi*	*HWB_Ratio*	*Ho*	*He*	*I*
Core	1.86	1.32	0.18	0.22	0.17	0.21	0.21	0.49
ALL	2	1.34	0.19	0.23	0.08	0.23	0.23	0.54

## Data Availability

The authors declare that the original data of this study are available from the corresponding authors.

## Supplementary Materials

The following supporting information can be downloaded at https://www.mdpi.com/article/10.3390/ani15182759/s1, Table S1: Quality summary of sequencing data; Table S2: Statistics of sequencing depth and coverage of all samples.
